# Supplementary material for: Minimal Change Disease Is Associated with Mitochondrial Injury and STING Pathway Activation
Source: J Clin Med. 2022 Jan 24;11(3):577. doi: 10.3390/jcm11030577 (PMC8836778; doi:10.3390/jcm11030577)
Supplement: Supplementary file 1 [file jcm-11-00577-s001.zip › jcm-1556358-SI.pdf]

Journal of
Clinical Medicine

MDPI

Supplementary Materials

Table S1. Baseline characteristics of the MCD and IgAN groups in the retrospective study.

Variable	MCD Group (n = 20)	IgAN Group (n = 20)	p-Value
Age (years)	43.5 ± 17.0	43.5 ± 16.4	>0.999
Sex (male)	13 (65.0)	13 (65.0)	>0.999
Body mass index (kg/m^2)	25.9 ± 3.5	25.9 ± 4.6	0.718
Hypertension	4 (20.0)	8 (40.0)	0.168
Systolic blood pressure (mmHg)	127.4 ± 11.0	130.2 ± 14.2	0.623
Diastolic blood pressure (mmHg)	82.1 ± 8.6	81.8 ± 7.7	0.940
Mean arterial pressure (mmHg)	97.2 ± 8.3	97.9 ± 8.4	0.821
Baseline eGFR (mL/min/1.73 m^2)	86.8 ± 20.6	66.1 ± 29.4	0.008
Baseline proteinuria (g/day)	5.65 ± 3.69	1.76 ± 1.60	<0.001
Use of ARB or ACE inhibitors	15 (75.0)	14 (70.0)	0.723

Data are presented as mean ± standard deviation for continuous variables and as number (%) for categorical variables. MCD, minimal change disease; IgAN, immunoglobulin A nephropathy; eGFR, estimated glomerular filtration rate; ARB, angiotensin II receptor blocker; ACE, angiotensin-converting enzyme.

Table S2. The type, duration, and cumulative dose of immunosuppressive agents administered during the follow-up period in patients with MCD in the retrospective study.

Treatment	High-Intensity Group (*n* = 6)	Low-Intensity Group (*n* = 14)	*p*-Value
Type of immunosuppressive agents			0.354
CS monotherapy (*n* = 7)	1 (16.7)	6 (42.9)	
CS + CYC or CS + CsA (*n* = 13)	5 (83.3)	8 (57.1)	
Duration of immunosuppressive treatment, month	76.6 ± 29.0	41.8 ± 38.3	0.019
Duration of CS treatment, month	55.6 ± 29.1	35.4 ± 39.6	0.111
Cumulative dose of CS (mg/kg)	416.6 ± 257.7	184.1 ± 119.7	0.061

Data are presented as mean ± standard deviation for continuous variables and as number (%) for categorical variables.
MCD, minimal change disease; CS, corticosteroid; CYC, cyclophosphamide; CsA, cyclosporine A.
